# Navigating parental hesitancy in public health: the case for RSV immunization in newborns

**DOI:** 10.1038/s41372-025-02282-5

**Published:** 2025-04-11

**Authors:** Michael K. Baumgartner, Gregor Hanslik, Mara Schneider, Manuela Franitza, Michael Schneider, Henrike Rieger, Hans-Christoph von Andrian, Melanie L. Conrad, Fabian B. Fahlbusch

**Affiliations:** 1 Neonatology and Pediatric Intensive Care, Faculty of Medicine, University of Augsburg, 86156 Augsburg, Germany; 2 Division of Neonatology and Pediatric Intensive Care Medicine, Department of Pediatrics and Adolescent Medicine, Friedrich-Alexander-University of Erlangen-Nürnberg, 91054 Erlangen, Germany; 3 Department of Obstetrics and Gynecology, Faculty of Medicine, University of Augsburg, 86156 Augsburg, Germany; 4 Department of Gynecology and Obstetrics, Friedrich-Alexander-University of Erlangen-Nürnberg, 91054 Erlangen, Germany; 5 Institute of Microbiology, Infectious Diseases and Immunology, Charité–Universitätsmedizin Berlin, Corporate Member of Freie Universität Berlin, Humboldt-Universität zu Berlin, 12203 Berlin, Germany

**Keywords:** Paediatrics, Preventive medicine

## Abstract

Respiratory syncytial virus (RSV) poses a significant risk to newborns, particularly during the first months of life, and remains a leading cause of pediatric hospitalization in Germany. Despite the approval of innovative immunoprophylaxis methods like nirsevimab, parental hesitancy persists, driven by knowledge gaps and concerns about safety. This study assesses the healthcare burden of RSV during the 2022/2023 and 2023/2024 seasons and explores parental perspectives on immunization just prior to the widespread availability of nirsevimab in late 2024. Data revealed a significant hospital burden, including rising intensive care admissions requiring respiratory support. A survey conducted in two Bavarian maternity wards found that while 78% of parents were aware of RSV, only 53% supported postnatal immunization. These findings highlight the groundbreaking potential of RSV immunization and underscore the urgent need for targeted communication strategies to address parental concerns to ensure successful implementation of these programs.

## Introduction

Respiratory syncytial virus (RSV) remains a significant threat to newborns, particularly those born premature or with underlying health conditions. However, the majority of severe RSV cases actually occur in previously healthy infants. Each winter, the increase in pediatric RSV cases places immense strain on healthcare systems, often pushing children’s hospitals to full capacity and leaving parents with limited care options. In the United States, RSV was the leading cause of bronchiolitis, resulting annually in approximately 2.1 million outpatient visits, up to 80,000 hospitalizations, and up to 300 deaths among children under five years old, based on prepandemic data from a 2009 study [1]. A recent cross-sectional study analyzing 288,816 children under five across 48 US pediatric hospitals revealed a significant postpandemic surge in RSV infections during the 2022–2023 season. Compared to prepandemic seasons, there was an 86.7% increase in hospitalizations and a 70% rise in children requiring advanced respiratory support, with patients being older and having fewer comorbidities [2]. These findings underscore the evolving trends in RSV severity and hospitalization needs, providing critical data to optimize vaccine distribution and guidelines for future RSV seasons.

RSV infections are also the most common cause of hospitalization in infants in Germany, especially in the first six months of life. A cross-sectional analysis of pediatric hospitals in Germany from October 2021 to January 2022 reported an average of 3.6 patients admitted per hospital per day to general wards and 0.4 patients to PICUs, with 11.5% requiring respiratory support; the majority of these patients were under two years of age [3]. Alarmingly, the number of patients admitted to ICUs for RSV during the 2022/2023 season nearly doubled compared to the previous year [4]. RSV accounted for the largest proportion of hospital and ICU admissions among children under five during the 2022/2023 respiratory infection season, with the lowest comorbidity rate (33.9%) among pathogens, highlighting that RSV can cause severe morbidity even in otherwise healthy infants [4].

In the light of this ongoing challenge, the German Standing Committee on Vaccination (STIKO) issued a prophylaxis with monoclonal antibodies as a standard recommendation [5] aiming at mitigating the severe consequences of RSV infection and reducing the burden on the healthcare system during peak seasons. As of June 27th, 2024, all newborns and infants should receive the monoclonal antibody nirsevimab (trade name: Beyfortus®) as a single dose before their first RSV season experienced (usually between October and March). Infants born between April and September should, if possible, receive it in the fall before the start of their first RSV season. Newborns born during RSV season should receive nirsevimab as soon as possible after birth, ideally upon discharge from the birthing center. In Germany, the STIKO recommends administration of nirsevimab at the standard pediatric well-child visit during birth hospitalization (3rd to 10th day of life), alike the statements given by the U.S. Centers for Disease Control and Prevention (CDC) and the American Academy of Pediatrics (AAP) in August of 2023 [4, 5]. Nirsevimab’s safety profile is well-established, building on decades of clinical experience with the related monoclonal antibody palivizumab (Synagis®), which has been safely administered to neonates at high risk of severe RSV disease since 1998. Unlike palivizumab, nirsevimab has been engineered to provide extended efficacy, lasting up to five months and covering an entire RSV season with a single dose [6, 7].

The STIKO’s recommendation serves as a pivotal anchor in the conversation about RSV prevention. Its implementation coincides with a period of heightened focus on improving neonatal and pediatric health, as highlighted by the legislative changes granting children a right to RSV immunization in Germany [8]. This underscores the groundbreaking nature of the intervention, which has the potential to significantly alleviate the healthcare and economic burden of RSV by reducing hospital admissions and intensive care needs.

During this transitional period, we conducted a dual-center survey to assess parental willingness for RSV immunization, with a focus on identifying actionable solutions to address barriers. Additionally, we performed a retrospective analysis of RSV case numbers at one of the participating centers to contextualize the burden of disease.

## Methods

The retrospective analysis of local epidemiologic trends in RSV-related healthcare burden included data on RSV infections from October to March during the 2022/2023 and 2023/2024 seasons, collected at the Department of Pediatrics and Adolescent Medicine, University of Augsburg, Germany. All patients with an RSV-related diagnosis, identified using the ICD-10 coding system, were included in the analysis. The total number of RSV-affected children was recorded for each month within the specified time frame. Exclusion criteria encompassed patients who did not have an RSV-related diagnosis based on ICD-10 codes or whose medical records lacked sufficient detail for accurate inclusion in the analysis. Additionally, duplicate entries, cases with incomplete data, or patients diagnosed outside the defined study period were excluded.

The survey was conducted at the German neonatal and obstetric units of the University hospitals Erlangen and Augsburg in August 2024. The inclusion criteria for the survey required participants to be parents of newborns receiving care on the maternal wards of the University hospitals Erlangen and Augsburg during August 2024. Participants needed to have sufficient proficiency in German to understand and respond to the survey. Participants were required to provide verbal agreement to participate in the survey, as no written consent was necessary due to the study’s anonymous design. Exclusion criteria included parents who declined to provide informed consent. Parents who were unable to complete the survey due to language barriers or cognitive impairments were excluded. To prevent duplicate data, parents who had already participated in the survey were also excluded. Additionally, parents unavailable due to acute medical or personal emergencies during the survey period were not included. The survey, provided as Supplementary Table S1, consisted of 12 questions addressing parents’ (1) awareness of RSV-related respiratory infections, (2) knowledge of prevention through prepartum vaccination and postpartum immunization, and (3) awareness of the recommendation for RSV antibody immunization on the third day of life.

### Institutional review board statement

All procedures were conducted in accordance with applicable institutional and national guidelines and regulations. Ethics approval and consent to participate were not required for this study, as no patient data were collected, and the survey was conducted anonymously. Therefore, this study does not fall under the scope of human subject research as defined by the relevant ethical guidelines and regulatory frameworks. Consequently, it qualified for a waiver of Institutional Review Board (IRB) review, as confirmed by the local Ethics Committee at the Friedrich-Alexander-Universität Erlangen-Nürnberg (#23-41-ANF).

## Results

### Local epidemiologic trends in RSV-related healthcare burden

**Fig. 1:**
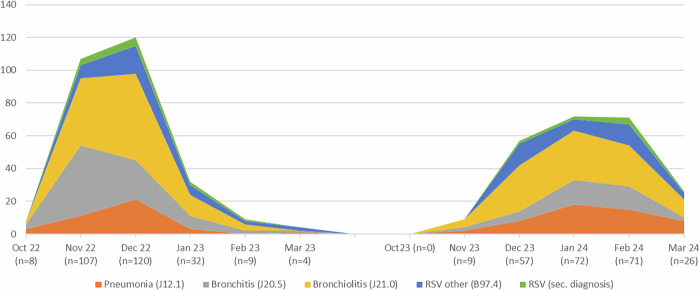
RSV infections from October to March 2022/23 and 2023/24 at the Dept. of Pediatrics and Adolescent Medicine, University of Augsburg, Germany. Legend: n = total number of RSV-affected children/month (y-axis); (ICD-10) provided with the respective diagnosis.

Our data indicate a total of 280 inpatient RSV cases recorded from October to March during the 2022/2023 season and 235 cases during the 2023/2024 season (Fig. 1). During these periods, 39.5% and 42.0% of the cases, respectively, were diagnosed with bronchiolitis. The maximum daily admission rates due to RSV were 3.9 patients in December 2023 and 2.5 patients in February 2024. PICU treatment was required for 7% of cases during both observational periods. 80% and 66.7% of these patients were treated with respiratory support for a median duration of 101 and 108 h, respectively.

### Survey results: understanding parental decisions in a Bavarian NICU setting

**Fig. 2:**
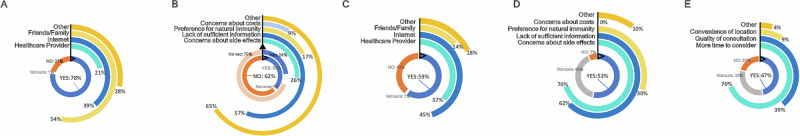
Parental Awareness, Knowledge, and Attitudes Toward RSV Immunization. **A** Awareness of respiratory infections caused by RSV; (**B**) knowledge of prepartum RSV vaccination; Legend: △*= If yes, were you vaccinated against RSV during pregnancy? ▲* = *If you were not vaccinated, why did you decide against it?* No vac = not vaccinated; Vac = vaccinated; (**C**) knowledge of postnatal RSV immunization; (**D**) consent to RSV immunization at 3rd day of life, and (**E**) preference for outpatient immunization. The figure utilizes circular bar graphs to represent survey responses, with the main answers to each question displayed in the center of the circles and detailed sub-questions further outside, evolving from △ = If yes, (…). Survey questions are detailed in Supplementary Table S1.

To gain deeper insights into parental perspectives on RSV immunization, we conducted a survey in August 2024 over one month. The survey was carried out in two tertiary birth clinics at university hospitals in Bavaria, Germany, shortly after the STIKO recommendation was published and just before the onset of the 2024 RSV season. The survey targeted parents post-delivery, focusing on their awareness of RSV, attitudes towards maternal vaccination, and their willingness to immunize their newborns with antibodies before discharge. The results are summarized in Fig. 2.

Our survey involved 87 parents, who provided insights into their knowledge of RSV and their views on preventive measures, representing a participation rate of 29% in both centers. A promising finding was that 78% of parents were already aware of RSV and its potential to cause severe respiratory illness in infants, via family and friends, as well as the internet as top sources of information (Fig. 2A). Health providers played a minor role (21%).

Only 37% of the participants were aware of the possibility for prepartum vaccination against RSV of whom only 24% got vaccinated. Reasons for not getting vaccinated were diverse, in many cases the option was not offered by their Obstetrician. Interestingly, 57% did not get vaccinated due to lack of information. Costs were a minor concern (Fig. 2B).

Furthermore, 59% knew about the option to immunize their newborns with monoclonal antibodies before leaving the hospital. Their knowledge mostly came from the internet and health providers (Fig. 2C). Only 53% of parents were willing to opt for postnatal immunization at day 3, with many citing concerns about the safety and necessity of the intervention and lack of information (Fig. 2D). 47% of parents would rather have their newborn immunized by their pediatrician hoping to have more time for consideration and expecting a more suiting form of consultation (Fig. 2E).

## Discussion

This study underscores the significant burden of RSV on healthcare systems, characterized by high rates of hospitalization and intensive care admissions. Our findings reveal notable parental hesitancy toward immunization primarily driven by knowledge gaps, concerns about safety / lack of trust and feelings of being rushed due to the timing shortly after birth. Our seasonal RSV data aligns with findings from cross-sectional studies from the U.S. [1, 2] and Germany [3, 4] underscoring the representativeness of our surveyed community.

While in the Netherlands, RSV-related PICU admission costs approximately 3.1–3.8 million € per season [9] for Germany Poshtiban et al. estimated the annual RSV-related health burden for the inpatient treatment of the pediatric population to be 66 million € [10]. These figures highlight the immense financial strain RSV places on healthcare systems, further reinforcing the importance of preventive strategies like immunization to reduce both clinical and economic burdens. As a consequence, the state of Bavaria has made efforts to sufficiently allocate its pediatric care resources via telecommunication [11] in order to maintain pediatric (intensive) care for non-infectious patients during RSV season [12].

### Opportunities to enhance parental acceptance of RSV immunization: lessons from Bavarian and U.S. surveys

Our survey included 87 parents from two tertiary NICU centers, making it significantly larger than the contemporaneous study by Hinderstein et al. in the United States, which encompassed 28 interviews at a single center [13]. Notably, their study employed a different methodological approach. Utilizing the Health Beliefs Model as a conceptual framework, their study identified actionable opportunities to influence perception-based factors such as susceptibility, severity, benefits, and barriers, as well as decision-making drivers like self-efficacy and cues to action. Consistent with our findings, Hinderstein et al. [13] demonstrated that parental acceptance of nirsevimab remains a critical challenge despite its official approval, partly shaped by negative perceptions linked to prior experiences with the COVID-19 vaccine and the miscommunication surrounding its rollout [14]. Additionally, they identified persistent barriers such as knowledge gaps, misinformation, and concerns about safety, with key issues including confusion between monoclonal antibodies and vaccines, as well as the influence of provider trust [13]. Noteworthy, trust in clinicians has long been recognized as a critical factor influencing vaccine decision-making and remains a highly influential determinant [15], however, the limited time available during the short stay after birth makes it challenging for pediatricians to establish a strong and healthy foundation of trust. In our study, parental acceptance of RSV immunization may have been positively influenced by providing sufficient information in advance and allowing more time for consideration, emphasizing the need to shift vaccination-related discussions earlier in the care continuum. This emphasizes the importance of implementing robust prenatal information campaigns addressing these barriers, particularly through obstetricians, to ensure parents are adequately informed before delivery.

While nearly 80% of parents in our study reported being informed about the impact of RSV in general, we did not assess the accuracy or depth of their knowledge, which represents a limitation of our study. However, rejection of immunization in our cohort was often linked to a clear knowledge gap and fears about potential side effects. Hinderstein et al. similarly found that some parents perceived nirsevimab as “new and understudied,” emphasizing the critical need for transparent communication about its safety profile, its similarity to established antibodies, and its rigorous clinical evaluation [13].

Such efforts would help to unlock the true potential of this intervention by reaching more children. This aligns with the groundbreaking potential of RSV immunization to transform pediatric intensive care by significantly reducing RSV-related hospital and NICU admissions.

### Leveraging media to build trust and combat misinformation

In times where parents commonly seek health-related information online, social media can positively influence vaccination-related behaviors during pregnancy. Unfortunately, it may also serve as a platform for spreading misinformation [16]. In this context, our recent analysis of German health information available online highlighted the diminished quality of content, partly driven by the growing trend of commercializing information [17]. In addition, recent efforts to maintain fact-checking on online platforms are being scaled back, even though our survey and the study by Hinderstein et al. [13] show that these platforms remain important sources of information for (future-to-be) parents and tools to counter potential misinformation [18]. Medical societies are uniquely positioned to leverage these media channels to disseminate accurate information, counter misinformation, and build trust by engaging a broad audience with authoritative and evidence-based guidance.

### The role of public health in shaping perceptions

Considering the safety aspects and in the international context, professional societies such as the German Society for Gynecology and Obstetrics (DGGG) support the recommendation to administer the maternal RSV vaccine from 32 to 36 weeks of pregnancy [19–22]. Currently, however, the maternal vaccine is not yet listed in the German Vaccination Guideline and is therefore not covered by general health insurance and reimbursement for private patients is not possible.

At the time of writing this article, the structural requirements (economic, insurance and medical personnel) to implement the newborn  immunization program comprehensively were under discussion with the Federal Ministry of Health in Germany and carried out in public [23–25]. At this time, the Association of Leading Pediatric and Adolescent Doctors in Germany (VLKKD) has estimated the need for a 25% part-time job for the immunization of 1000 births in the period from October to March, with an investment of €300-350,000 for the vaccine and approximately 20 min per immunization including informative conversation, patient consent and documentation [26].

## Summary

Our study shows that apart from the structural requirements, the success of this public health initiative strongly depends on parental acceptance. Insights from recent international findings reveal that parental concerns about RSV immunization often stem from misinformation and a lack of trust in healthcare providers. Addressing these barriers is critical for ensuring widespread adoption. Sustaining and possibly increasing the momentum of public health campaigns remains essential, particularly as immunization programs move from feasibility discussions to widespread implementation.

## Conclusion

The recent RSV seasons have underscored the critical importance of preventive strategies in safeguarding infants and managing the significant healthcare burden caused by respiratory infections. The STIKO’s recommendation to immunize newborns with monoclonal antibodies against RSV represents a landmark advancement in pediatric healthcare. However, the success of this initiative will depend on overcoming challenges such as healthcare structural barriers and parental hesitancy.

Parental concerns, fueled by misinformation and a lack of trust in healthcare providers, remain key obstacles. Addressing these barriers requires a multipronged approach involving early counseling, targeted public health campaigns, and ongoing collaboration between healthcare providers, policymakers, and public health authorities. Clear, consistent communication tailored to parental needs is vital for ensuring widespread acceptance and understanding of RSV immunization programs.

The legislative changes granting children the right to RSV immunization in Germany reflect a pivotal moment in public health policy, highlighting the urgency of effective program implementation to fully realize the transformative potential of this groundbreaking intervention. By addressing both systemic challenges and parental concerns, this initiative has the potential to significantly reduce the seasonal burden on healthcare systems and protect vulnerable newborns from the severe consequences of RSV.

While the immunization program is now in place, this study provides a critical foundation for future research evaluating its real-world impact. Follow-up studies should assess the program’s influence on hospitalization rates, PICU admissions, and healthcare system strain, as well as explore changes in parental acceptance over time. Such research is essential for optimizing RSV immunization strategies, ensuring equitable access, and informing public health policies globally.

## Supplementary information

Supplementary Table S1
